# 3D Bioprinting Techniques and Bioinks for Periodontal Tissues Regeneration—A Literature Review

**DOI:** 10.3390/biomimetics9080480

**Published:** 2024-08-09

**Authors:** Nátaly Domingues Almeida, Camila Alves Carneiro, Andrea Carvalho de Marco, Vinicius Carvalho Porto, Rodrigo França

**Affiliations:** 1 Department of Diagnosis and Surgery, University of the State of São Paulo, São José dos Campos 12200-000, SP, Brazil; nataly.d.almeida@unesp.br (N.D.A.); andrea.marco@unesp.br (A.C.d.M.); 2 Dental Materials Research Lab, Department of Restorative Dentistry, Dr. Gerald Niznick College of Dentistry, Rady Faculty of Health Sciences, University of Manitoba, Winnipeg, MB R3E 0W2, Canada; camila-alves@usp.br; 3 Department of Prosthodontics and Periodontics, Dental School of Bauru, University of São Paulo, Bauru 17000-000, SP, Brazil; vcporto@fob.usp.br

**Keywords:** periodontal ligament, bioprinting, tissue engineering

## Abstract

The periodontal tissue is made up of supporting tissues and among its functions, it promotes viscoelastic properties, proprioceptive sensors, and dental anchorage. Its progressive destruction by disease leads to the loss of bone and periodontal ligaments. For this reason, biomaterials are constantly being developed to restore tissue function. Various techniques are being used to promote regenerative dentistry, including 3D bioprinting with bioink formulations. This paper aims to review the different types of bioink formulations and 3D bioprinting techniques used in periodontal tissue regeneration. Different techniques have been formulated, and the addition of different materials into bioinks has been conducted, with the intention of improving the process and creating a bioink that supports cell viability, proliferation, differentiation, and stability for periodontal tissue regeneration.

## 1. Introduction

The periodontium is made up of the supporting tissues of the teeth, which include the cementum, the periodontal ligament, the gingiva, and the alveolar bone [1]. The periodontal ligament is a connective tissue composed mainly of bundles of collagen fibers [2], promoting viscoelastic properties, proprioceptive sensors, and dental anchorage. They are also involved in tissue regeneration and alveolar bone homeostasis [1]. Periodontal disease is an inflammation of the supporting tissues, in which there is a progressive destructive change that leads to the loss of bone and periodontal ligaments [3]. 

For this reason, biomaterials are constantly being developed with the aim of restoring tissue function. Various techniques have been used to promote regenerative dentistry, including 3D bioprinting, which is considered to be an innovative technological approach. Thus, growth factors, viable cells, biomaterials, and bioactive molecules are used to create tissues and organs, and when these elements are involved, they are referred to as bioinks [4,5]. 

There is a wide variety of bioinks, but the most-used ones are based on the following materials: agarose, alginate, collagen, and hyaluronic acid [6]. They mimic the extracellular matrix of the target tissue, promoting proliferation, cell differentiation, and controlled biodegradability [7]. Thus, the application of stem cells is fundamental, especially in the oral cavity, where oral tissues are rich in sources of stem cells and are therefore important in the regeneration or replacement of damaged or diseased tissues [8,9,10]. 

Periodontal ligament stem cells and gingival mesenchymal stem cells can promote periodontal regeneration [10,11]; therefore, they can be used in the 3D bioprinting technique. However, gingival mesenchymal stem cells show greater proliferation, differentiation, and bone formation in vivo [11,12]. In light of this, periodontal regeneration consists of the restoration of various tissues and is considered a challenge in clinical practice.

Therefore, this review aims to review bioinks composed of different types of materials, evaluating their results and limitations when used in the regeneration of supporting tissues, mainly the periodontal ligament.

### 1.1. Methodology and PRISMA Flowchart

This study comprises a literature review based on the following research question: ‘What are the types of bioink formulations and 3D bioprinting techniques used in the regeneration of periodontal tissues, and what are the benefits demonstrated by these approaches in promoting tissue regeneration?’ Therefore, this study aimed to investigate the benefits of this specific technique for periodontal tissue regeneration.

#### 1.1.1. Search Strategy

**Figure 1:**
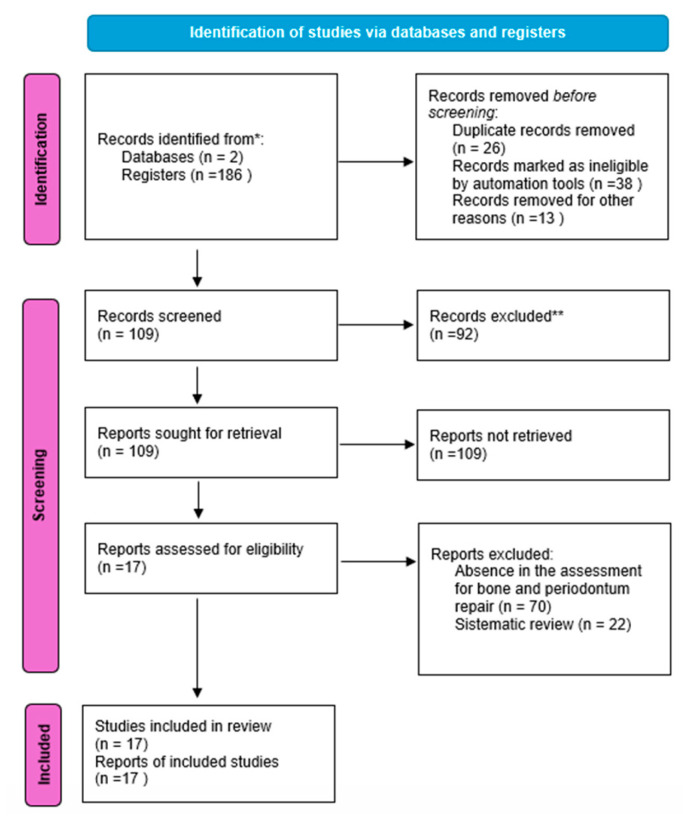
PRISMA flowchart.

To conduct this study, the following combinations of keywords were used: “bioink”, “bioprinting”, “periodontal ligament”, “periodontal reconstruction”, “bone regeneration”, and “reconstructions”, with “and” as an integrative connector for the searches, using the PubMed and Scielo databases. The searches were conducted in March and April 2024. The general distribution of the selection process for the articles is illustrated in Figure 1; for this, the PRISMA Flowchart template was used. The “*” and “**” icons in the flowchart blocks indicate the level of importance or significance of the corresponding step or decision. The single asterisk (*) typically denotes a moderate level of importance, while the double asterisks (**) indicate a higher level of importance or priority.

#### 1.1.2. Inclusion Criteria

Studies involving both in vitro and in vivo research, as well as those focusing specifically on 3D bioprinting techniques rather than other printing techniques or regenerative approaches, were selected.

#### 1.1.3. Exclusion Criteria

Systematic reviews and literature reviews were excluded. Additionally, studies lacking statistical analyses or utilizing bioprinting techniques other than 3D printing were also eliminated.

#### 1.1.4. Results

After conducting an extensive search using specific descriptors, a total of 186 articles were initially identified. Of these, 109 articles were deemed potentially eligible for further review. However, 26 articles were found to be duplicates and were subsequently removed from the pool. Additionally, 38 studies were excluded from consideration because they did not pertain to the relevant topic under investigation. Upon closer examination of the remaining articles, 92 studies were excluded for several reasons. A significant number of these studies did not include an assessment of bone and periodontium repair, which was a critical criterion for inclusion in this review. 

## 2. 3D Bioprinting Techniques

The objective of additive manufacturing is to reproduce tissues and organs similar to native ones, with the purpose of repairing them. This technique demonstrates the ability to produce high-precision scaffolds, allowing detailed control of cell behavior [13]. Several areas are benefiting from the development of the 3D technique, including neural tissue engineering, pharmacology, oncology, and primarily periodontal regeneration, particularly highlighted for its application potential. Table 1 summarizes different applications of 3D bioprinting for the treatment of periodontal disease.

Furthermore, 3D structures are fabricated through computational and computer-aided design [7]. These structures involve several steps, methods, and forms of printing, such as inkjet, laser-assisted, extrusion, microextrusion, and digital light processing. These methods allow the creation of complex and precise models, suitable for different tissue engineering needs and other biomedical applications.

The inkjet approach is characterized by droplets and demonstrates relatively high cell viability [14,15,16]. This method can be generated by various systems such as acoustic, piezoelectric, hydrodynamic, electrostatic, thermal processes, and microvalves [7]. They are like commercial printers and therefore have a low cost [15]. 

On the other hand, laser-assisted printing is considered a complex and expensive method, which differs from the inkjet technique. In addition, laser-assisted printing demonstrates high-resolution, using bioink with greater viscosity and superior cell viability, because there is no direct contact between the bioink and the dispenser, so there is no mechanical stress on the cells [17]. However, it is a little-used technique that requires more research to standardize the sizes of the droplets generated.

Extrusion is the most widely used method and is based on microextrusion, which is characterized by controlled deposition, layer by layer. In addition, it is possible to print a variety of materials because it is applied employing a continuous force and not in droplets [18,19], for this reason, the cells are exposed to greater stresses, thus decreasing cell viability [15,20].

Digital light processing is laser-assisted and differs from the inkjet and extrusion techniques in that it is nozzle-based. As such, this technique presents some changes in terms of resolution and material selection [21,22,23]. Also, light-sensitive biomaterials cannot be used in this method due to the photopolymerization process that is generated by ultraviolet light [24].

## 3. Bioink

Bioink is considered essential for 3D bioprinting and is classified into two categories: scaffold-free bioinks, which can include tissue threads, cell pellets, and tissue spheroids; and scaffold-based bioinks, which can contain decellularized matrix, cell aggregates, microcarriers, and hydrogels [25,26]. Both types need to be biocompatible to act as cellular transporters and thus promote the regeneration of new tissues. An ideal combination of physical properties is also required, i.e., having strength and elasticity to replicate the mechanical properties of the target tissue [27]. Furthermore, it is crucial to have good viscosity to ensure adequate printing and reproducibility. Therefore, material selection should be based on the target tissue to be constructed [27].

A variety of materials can be classified as natural or synthetic and used as bioinks [25]. Natural materials can be modified, and most of them have good compatibility and high cellular activity, which is why they are widely used and can be applied to different types of tissues, including bone tissue [26]. Synthetic materials are produced through chemical synthesis, so their properties are controllable and can be tailored to specific needs. Because of this, they can exhibit high tensile strength [27] and, consequently, can demonstrate greater advantages in bone regeneration [28,29,30,31].

### 3.1. Bioink and Alveolar Bone Regeneration

Repairing damage to the alveolar bone is still a challenge for dentists, which is why bone tissue engineering seeks to develop innovative techniques to promote proper repair. Thus, Gao et al., 2023 [28] analyzed a bioink containing synthetic and natural materials: polyethylene glycol diacrylate, Pluronic F127 diacrylate, and gelatin methacrylate. As shown in Table 1, their study showed good results both in vitro and in vivo when compared to a bioink composed only of synthetic materials. 

However, this result differs from the study by Gungor-Ozkerim et al., 2018 [29] because, according to them, most natural polymers have low mechanical properties, reducing their reliability in the printing procedure and their ability to regenerate hard tissues such as bone. The study of Gao et al., 2023 [28], has shown that a combination of materials can provide better results. Furthermore, in a randomized clinical study of Bajestan et al., 2017 [32], observed that stem cell therapy induces bone regeneration of small defects and is therefore a great candidate for use in bioinks.

Another strategy was addressed for some authors; they used bioinks composed of hyperplastic bones loaded with nanoparticles superparamagnetic iron oxide nanoparticles, and their results were satisfactory (Table 2). There are a variety of nanomaterials that can also prove this biofunctional behavior [31,32], being classified as follows: carbon-based nanoparticles [33]; natural and synthetic biopolymer nanoparticles [24,30,34]; metallic nanoparticles [35,36,37]; and ceramic nanoparticles [38,39,40,41,42,43].

A study carried out by Orozco et al., 2022 [43], aimed to develop a nanocomposite bioink comprising strontium–carbonate nanoparticles and gelatin–methacryloyl, using magnesium hydroxide nanoparticles scaffolds and polycaprolactone as bioactive internal reinforcements, which has shown positive implications in bone regeneration, improving the deposition of specific bone matrix; thus, we can conclude that the insertion of nanoparticles is considerably promising in bone repair.

As shown in Table 1, Cao et al., 2023 [44], used a bioink composed of a synthetic nano-clay, laponite. Nano-clays are therefore classified as nanomaterials. The study by Hafezi et al., 2023 [45], added various concentrations of nano-clay to gelatin methacrylate, alginate, and nano-clay with the intention of modifying viscosity [45]. The results obtained with the addition of 2% nano-clay revealed better physical, mechanical, and biomedical properties, making them excellent candidates for repairing tissue and cartilage defects.

**Table 1:** Applications of 3D bioprinting in periodontal disease.

Application	Biomaterials	Cell Source	Bioprinting	Properties	Reference
Increase the quality and quantity of keratinized gingiva	Gelatin and sodium alginate	Gingival fibroblasts	3D printing	Greater promotive effect on soft tissue regeneration	[46]
Regeneration of the oral soft tissue	Gelatin, sodium alginate, and platelet-rich fibrin	Multipotent adult stem cells	Extrusion-based 3D printing	Exhibited mechanical and rheological properties; excellent biocompatibility in vitro and in vivo	[47]
Formation of cementum	Polycaprolactone, oly)lactic-co-glycolic acids) and connective tissue growth factor	Periodontal ligament stem/progenitor cells	Layer-by-layer 3D printing	Can guide the formation of a fully integrated periodontium-like structure, consisting of cementum/dentin, periodontal ligamen, and alveolar bone in vivo	[48]
Guided bone regeneration in peri-implants	Polycaprolactone, poly(lactic-co-glycolic acid), β-tricalcium phosphate, and collagen	Gingival fibroblast	Extrusion-based 3D printing	The 3D-printed PCL/PLGA/β-TCP membrane was confirmed to have substantial efficacy as a resorbable GBR membrane for peri-implant defect treatment	[49]

PCL: polycaprolactone; PLGA: poly (lactic-co-glycolic acid); *β*-TCP: *β*-tricalcium phosphate.

**Table 2:** Regenerating alveolar bone with bioink.

Study	Bioink Composition	Cell Source	Bioprinting	Target Tissue	Antibacterial Results	In Vitro Results	In Vivo Results
Yu et al., 2024 [50]	Epsilon-poly-L-lysine hydrogels modified with glycidyl methacrylate	Myeloid-derived suppressor cells and periodontal ligament stem cells	Digital light processing printer	Multifunctional and biomimetic alveolar bone	EPM was shown to effectively kill the periodontopathic bacteria depending on the natural antibacterial properties of the EPL; MDSCs-MV effectively killed the periodontopathic bacteria	1. Control 2. MDSCs 3. MDSCs-MV MDSCs-MV play an anti-inflammatory and mineralizing role in osteoblast derived from periodontal ligament stem cells.; additionally, could significantly enhance the mineralizing capacity of PDLSCs-derived osteoblast	1. Control (no scaffold + no bacteria) 2. Scaffold + P.g intervention 3. EPLGMA scaffold + P.g intervention 4. EPLGMA/MDSCs-MV scaffold + P.g intervention 5. EPM scaffold + P.g intervention Micro-CT and histological staining demonstrated that the EPM scaffold similarly had an excellent anti-inflammatory and bone regeneration efficacy
Miao et al., 2023 [47]	Gelatin methacryloyl, sodium alginate bioactive glass microsphere	Mouse bone marrow mesenchymal stem cells and growth factors (BMP2 and PDGF)	Extrusion, layer-by-layer application	Bioactive scaffolds for simultaneous repair of periodontal bone and soft tissue	Not mentioned	1. GelMA/SA 2. GelMA/SA/BGM The incorporating bioactive glass into GelMA/SA hydrogel could improve its bioactivity and showed enhanced osteogenic differentiation and soft tissue repair capabilities in BMP2- and PDGF-loaded scaffolds	1. Not implanted 2. GelMA/SA/BGM 3. GelMA/SA/BGM 4. Containing cell-laden BMP2 and PDGF BMP2/PDGF 5. GelMA/SA/BGM scaffold containing BMP2 and PDGF Significant regeneration of gingival tissue, periodontal ligament, and alveolar bone was detected
Gao et al., 2023 [31]	Polyethylene glycol diacrylate, pluronic F127 diacrylate, and gelatin methacryloyl	Not mentioned	Digital light processing	Scaffold	Not mentioned	1. Normal medium 2. GPF 3. PF GPF facilitated the adhesion and proliferation of cells and effectively promoted the osteogenic differentiation of mesenchymal stem cells in an osteo-inductive environment	1. GPF 2. PF 3. Blank GPF possesses a satisfactory porous structure and mechanical properties to promote osteogenic differentiation under osteo-inductive conditions and guides bone
Cao et al., 2023 [44]	Methacrylated gelatin, methacrylated alginate, and laponite	Rat platelet-rich plasma	Layer-by-layer	Bone repair scaffold	Both sets of hydrogels containing PRP promoted macrophage M2 polarization	1. GA 2. PRP-GA 3. PRP-GA@Lap	1. GA/PCL 2. PRP-GA 3. PRP-GA@Lap/PCL 4. Blank control
						PRP-GA@Lap promoted the proliferation, migration, and osteogenic differentiation of rat bone marrow mesenchymal stem cells, accelerated the formation of endothelial cell vascular patterns	PRP-GA@Lap promoted vascular inward growth and enhanced bone regeneration at the defect site.
Miau et al., 2016 [51]	Periodontal ligament stem cells (PDLSCs)/methacrylate gelatine (GelMA) hydrogel	Periodontal ligament	Extrusion	Alveolar bone cells	Not mentioned	1. PDLSC with 3% GelMA 2. PDLSC with 5% GelMa 3. PDLSC with 10% GelMA	
						10% GelMA bioprinted constructs showed lower cell viability, less cell spreading.	10% GelMA bioprinted showed lower cell survival, but good bone regeneration
Shokouhimehr et al., 2021 [33]	Hyperelastic bone ink and oxide nanoparticles	Not mentioned		Cylindrical scaffold and disc scaffold	Incorporating 200 µg/mL of SPIONs increased antibacterial activity in comparison to the 60 µg/mL SPION-loaded group	1. SPION-free 2. SPION-loaded 60 µg/mL 3. SPION-loaded 200 µg/mL 60 µg/mL SPION-loaded group appeared to keep higher viability and exhibited greater potential for osteogenesis and mineralization	1. Control 2. SPION-loaded 60 µg/mL 60 µg/mL SPION-loaded HB scaffolds grafts show rapid integration with host tissue, ossification, and growth of new bone

EPM: glycidyl methacrylate modified epsilon-poly-L-lysine hydrogels bioink loading periodontal ligament stem cells and myeloid-derived suppressive cells membrane vesicles; EPL: Epsilon-poly-L-lysine; MDSCs-MV: myeloid-derived suppressive cells; MDSCs: myeloid-derived suppressive cells; PDLSCs: periodontal ligament stem cells; Pg: porphyromonas gingivali; EPLGMA: glycidyl methacrylate-modified epsilon-poly-L-lysine hydrogels bioink; BMP2: bone morphogenetic protein 2; GelMA/SA: methacryloyl bioactive sodium alginate; GelMA/SA/BGM: gelatin methacryloyl bioactive sodium alginate glass microspheres; GPF: gelatin methacryloyl Polyethylene glycol diacrylate pluronic F127 diacrylate; PF: polyethylene glycol diacrylate and pluronic F127 diacrylate; GA: methacrylated gelatin methacrylated alginate.

### 3.2. Bioink and Periodontal Ligament Regeneration

Due to its complexity, periodontal tissue regeneration involves the restoration of multiple types of tissues. In order to perform this process, several 3D printing techniques are used, like the freeform reversible embedding of suspended hydrogels (FRESH) used by Lin et al., 2021 [52] with, a bioink containing natural material, type I collagen to create a collagen-based microfiber scaffold [52]. The results (Table 3) show that the periodontal ligament cells were successfully seeded, and the cytoskeleton expansion, adhesion, and viability were good.

On the other hand, Tian et al., 2021 [53], created a mix of synthetic and natural materials as a bioink, using hydrogel, hydroxyapatite nanoparticles, and periodontal ligament stem cells [53]. This bioscaffold caused an effective stimulation of cell survival, proliferation, and differentiation, and also improved the mechanical properties and swelling capability. And, as shown in Table 1, Zhu et al., 2023 [54], also used periodontal ligament stem cells in GelMA hydrogel in three different concentrations (3%, 5%, and 10%) and the 10% of GelMa showed lower cell viability and cell survival; however, the addition of periodontal ligament stem cells contributed to the formation of new cells [54].

Nanotechnology was also used by Vurat et al., 2020 [55], through adding hydroxyapatite nanoparticles in a GelMa structure, which showed a high level of compressive strength of the periodontal tissue [55]. And Zhang et al., 2022 [56], the approach was used with nano-hydroxyapatite and bone marrow-derived mesenchymal stem cells in alginate/gelatin to improve the regeneration in the alveolar process; the nanoparticles increased the viscosity of the tissues and provided a slow degradation rate [54]. As previously mentioned, the nanoparticles are promising in bone repair due to osteoconductive properties, but also contribute to soft tissue regeneration [46,47,48,49,56,57,58,59].

To improve tissue engineering scaffolds for periodontal tissue regeneration using hydrogel, Miao et al., 2023 [47], developed a new one containing bioactive glass microsphere (BGM), and also applied it as a bio-ink to load with mouse bone marrow mesenchymal stem cells and growth factors for the fabrication of a scaffold for periodontal tissue regeneration [47]. As a result, a fully reconstructed periodontal structure was established in eight weeks post-transplantation; this could be due to BGM soft tissue repair ability that improved the physicochemical and biological properties of the hydrogel.

Another study that aimed to improve the GelMA hydrogel was developed by Yang et al., 2023 [48], through adding porcine dental follicle-derived decellularized extracellular matrix (dECM) to promote fibrogenesis and osteogenesis; it was shown to have the capacity to recreate the intrinsic native tissues, mimicking some tissues’ compositions and enhancing stem cell differentiation [44]. The results show (Table 3) that the addition of dECM improved the mechanical property and bioactivity of the hybrid bioink, which contributes to cell survival, biological behaviors, and tissue remodeling.

**Table 3:** Regenerating the periodontal ligament with bioink.

Study	Bioink Composition	Cell Source	Bioprinting	Target Tissue	Antibacterial Results	In Vitro Results	In Vivo Results
Yang et al., 2023 [48]	Methacrylate gelatin/decellularized extracellular matrix	Porcine dental follicles	Digital light projection	Periodontal module comprising periodontal ligament and module alveolar bone	Not mentioned	1. GelMA 2. GelMA + 5dECM 3. GelMA + 10dECM The periodontal module did not give rise to apparent immunological rejection or systemic damage	GelMA + dECM promoted the regeneration of functional periodontal tissues, higher alveolar bone recovery, more mature periodontal ligament fibers, and a more sophisticated fusion of the interface. GelMA + 5dECM promoted the regeneration of hybrid periodontal tissues, especially the anchoring structures of the bone–ligament interface, well-aligned periodontal fibers, and highly mineralized alveolar bone; GelMA, forming GelMA/dECM cell-laden bioink
Zhang et al., 2022 [56]	Alginate/gelatin nanohydroxyapatite	Gingival fibroblast cells and bone-marrow-derived mesenchymal stem cells	Extrusion	Not mentioned		1. GFs in AG 2. BMSC in AGH The viability of GFs in AG was higher than in BMSC AGH	1. AG 2. AGH (acellular) 3. AG + GFs/AGH + BMSCs (cell printed) The cellular printed construct displayed a more integrated structure and was better
Lin et al., 2021 [52]	Type 1 collagen-based	Periodontal ligament	Extrusion	Periodontal tissue	Not mentioned	Collagen-based microfibers were successfully fabricated; exhibited an enhanced tendency to promote healing and regeneration	Not mentioned
Raveendran et al., 2019 [60]	Gelatin methacryloyl and phenyl-2,4,6- trimethylbenzoylphosphinate	Human primary periodontal ligaments cells	Extrusion	Periodontal tissue	Not mentioned	1. Control casted cellular solution in GelMA 2. Casted cellular solution in GelMA with 0.05% LAP 3. Casted cellular solution in GelMA with a 20 s UV exposure 4. Casted cellular solution in GelMA with 0.05% LAP and with a 20 s UV exposure The bioprinted cellular GelMA (Printed) without LAP or UV irradiation; the cell viability of the 3D-printed PDLCs decreased	
Lee et al., 2014 [61]	Polycarprolactione- hydroxylapatite	From dental stem/progenitor cells	Layer-by-Layer	Region-specific micro-scaffolds	Not mentioned	1. PDLSCs 2. DPSCs 3. ABSCs Distinctive tissue phenotypes were formed with collagen I-rich fibers especially by PDLSCs and mineralized tissues by DPSCs, PDLSCs, and ABSCs	1. Control–DPSC with microspheres 2. DPSC-seeded with amelogenin 3. DPSC-seeded with BMP2 4. DPSC-CTGF DPSC-seeded multiphase Scaffolds upon implantation yielded aligned PDL-like collagen fibers inserted DSPP^+^, CEMP1^+^ mineralized matrix on one side and bone-like tissue on another side, which together recapitulated a putative periodontium complex

GelMA + dECM/GelMA + 5dECM/GelMA + 10dECM: methacrylate added gelatin derived from porcine dental follicles: alveolar bone modulus; GFs: gingival fibroblast cells; BMSCs bones: connective tissue growth factor; PDL: periodontal ligament; DSPP^+^: dentin sialophosphoprotein-positive; CEMP1^+^: cementum matrix protein 1-positive.

## 4. Future Insights into Bioprinting

Several biomaterials and techniques are being developed with the aim of improving osteoinduction, seeking a balance between mechanical resistance and osteogenic properties. However, numerous limitations have been identified, requiring investigations and approaches to enhance these properties. 3D bioprinting faces several significant challenges, often related to the printing method, the choice of bioinks, biomaterials, and cells. Recently, techniques such as 4D and 5D bioprinting have gained prominence in research.

4D bioprinting uses intelligent materials that change through external stimuli and are capable of altering their shape over time [50,60]. This can be achieved by coding these materials with 3D printing. This technique is considered recent, but it can promote significant effects in bone tissue engineering, as 4D-printed scaffolds are expected to adjust precisely to the geometry of the bone defect over time, thus facilitating dynamic bone remodeling [61,62].

Miao et al. (2016) [51] produced porous scaffolds with surface structures of polymerized soybean oil epoxidized acrylate and demonstrated potential for 4D applications due to the excellent cytocompatibility and memory effect. Furthermore, advanced cytotoxicity tests and adhesion to human bone marrow mesenchymal stem cells demonstrate progress in relation to biomaterials that can contribute to the success of this technique.

Several medical areas can benefit from these innovations, including vascular constructions, organs, and the nervous system [63]. However, studies with 4D-printed scaffolds are limited, necessitating the development of new “self-performing” biomaterials [61].

Research progress and the intention of improvement in additive manufacturing have led to the development of 5D and 6D printing. 5D printing has more robust mechanical properties, making it a promising technique for bone components. It stands out compared to 3D printing due to the formation of curved surfaces [51,64,65]. In turn, 6D printing combines 4D and 5D printing techniques, offering not only greater mechanical resistance but also the ability to respond to external stimuli [65]. Therefore, it is possible to obtain a scaffold with better projection and intelligent behavior [51]. Although 6D printing is not commonly used, it is expected to be costly; however, it can have a transformative impact on bone tissue engineering [66,67,68].

Despite several limitations, each technique has unique characteristics that can improve people’s quality of life, being considered innovative and revolutionary. Research needs to be carried out to further evaluate the long-term benefits and constraints, as well as develop designs and software to make these techniques more sophisticated and economically accessible. 

## 5. Discussion

The present review has examined the latest advancements in 3D bioprinting techniques and bioink formulations for periodontal tissue regeneration. The findings underscore significant progress in the field, highlighting both the potential and the challenges associated with these innovative approaches [1,6,7,8,9,10].

The evolution of 3D bioprinting has enabled the precise fabrication of scaffolds and tissue constructs that closely mimic the native architecture of periodontal tissues. Techniques such as inkjet, laser-assisted, extrusion, microextrusion, and digital light processing have been pivotal in achieving high-resolution and high-precision bioprinting. Each method offers unique advantages. For instance, laser-assisted printing provides high resolution and cell viability, while extrusion-based methods allow for the use of a wide variety of materials. However, each technique also presents specific limitations, such as the high cost and complexity of laser-assisted printing and the mechanical stress imposed on cells during extrusion [13,14,15].

The development of bioinks has been central to the success of 3D bioprinting in periodontal regeneration. The review highlights the diverse range of materials used in bioink formulations, including natural polymers like collagen and hyaluronic acid, and synthetic materials such as polyethylene glycol diacrylate. Natural materials are favored for their biocompatibility and ability to promote cellular activities, while synthetic materials offer controllable mechanical properties. The combination of these materials has shown promising results in enhancing the physical and biological properties of the bioinks [25].

The regeneration of periodontal tissues, particularly the periodontal ligament and alveolar bone, remains a significant challenge. The review presents various studies that have explored different bioink compositions and 3D printing techniques to address this issue. For example, the use of periodontal ligament stem cells and gingival mesenchymal stem cells in bioinks has shown potential in promoting tissue regeneration. Additionally, the incorporation of nanoparticles, such as hydroxyapatite, has been found to enhance the mechanical properties and osteogenic potential of the bioinks [25,26,27].

Despite the promising advancements, several challenges persist. The high cost of 3D bioprinting technology and the need for specialized bioinks tailored to specific tissues are significant barriers to widespread clinical application. Moreover, the long-term stability and functionality of bioprinted tissues require further investigation through extensive in vitro and in vivo studies [20].

The emergence of 4D and 5D bioprinting techniques offers exciting prospects for the future. These advanced methods, which involve the use of smart materials capable of changing shape in response to external stimuli, have the potential to further enhance the adaptability and functionality of bioprinted scaffolds. However, these technologies are still in their infancy and require substantial research to fully realize their potential [51,63,64,65,69,70].

## 6. Conclusions

In this review, we briefly investigate the use of bioprinting as a treatment for tissues lost due to periodontal disease, such as bone, periodontal ligament, and cementum. We analyze the challenges and contributions of this technique in the field of periodontics. Due to its high cost, access to this technology is restricted. Furthermore, the development of bioinks is a challenge, as it is necessary to use specific compounds for each target tissue. In this context, we highlight the efforts made to develop new types of bioinks, reformulating techniques or adding different materials with the aim of improving resistance, viability, proliferation, and cellular stability for periodontal tissue regeneration.

We conclude that bioprinting brings several benefits to periodontal regeneration, especially concerning bone defects, and is considered a revolutionary method. New techniques, such as 4D, 5D, and 6D bioprinting, have promising potential in bone repair. However, more in vitro and in vivo studies, as well as clinical approaches in humans, are needed to fully validate these techniques.
